# A Novel *Trans* Conformation of Ligand-Free Calmodulin

**DOI:** 10.1371/journal.pone.0054834

**Published:** 2013-01-29

**Authors:** Veerendra Kumar, Vishnu Priyanka Reddy Chichili, Xuhua Tang, J. Sivaraman

**Affiliations:** Department of Biological Sciences, National University of Singapore, Republic of Singapore, Republic of Singapore; Indian Institute of Science, India

## Abstract

Calmodulin (CaM) is a highly conserved eukaryotic protein that binds specifically to more than 100 target proteins in response to calcium (Ca^2+^) signal. CaM adopts a considerable degree of structural plasticity to accomplish this physiological role; however, the nature and extent of this plasticity remain to be fully understood. Here, we report the crystal structure of a novel *trans* conformation of ligand-free CaM where the relative disposition of two lobes of CaM is different, a conformation to-date not reported. While no major structural changes were observed in the independent N- and C-lobes as compared with previously reported structures of Ca^2+^/CaM, the central helix was tilted by ∼90° at Arg75. This is the first crystal structure of CaM to show a drastic conformational change in the central helix, and reveals one of several possible conformations of CaM to engage with its binding partner.

## Introduction

Calmodulin (CaM), a 16.7 kDa protein found in all eukaryotic cells, has been extensively studied as a primary calcium (Ca^2+^)-binding protein [1]. CaM mediates various processes, including inflammation, metabolism, apoptosis, smooth muscle contraction, intracellular movement, short-term and long-term memory, nerve growth, cell motility, growth, proliferation and the immune response [2]. The function of CaM is affected by post-translational modifications, such as phosphorylation, acetylation, methylation and proteolytic cleavage [3]. CaM consists of two homologous (46% sequence identity) globular lobes; each has a pair of Ca^2+^ binding sites (EF-hand motifs), connected by a flexible linker [4]. Each EF-hand motif comprises two α helices connected by a 12-residue loop (helix-turn-helix) and provides a suitable electronegative environment for Ca^2+^ ion coordination. The helices of two EF-hands motifs create a Phe and Met-rich hydrophobic pocket that is exposed to solvent and involved in target binding [5]. In *apo* CaM (Ca^2+^ free), the α-helices in the EF-hand motifs are positioned almost parallel to each other (closed conformation), whereas in the presence of Ca^2+^, the α-helices of the EF-hand motifs change their position relative to each other, forming an almost perpendicular conformation (open conformation) [6], [7]. Ca^2+^ therefore induces a large conformational change, exposing the hydrophobic surface and facilitating binding between Ca^2+^/CaM and a number of basic amphiphilic helices on target proteins [8]. The central helix of CaM is highly flexible and is the key to its ability to bind a wide range of targets [9].

Conformational flexibility plays a key role in CaM function. CaM is able to adopt a wide variety of conformations for its interaction with different targets [10], [11], [12], [13], [14]. The N- and C-terminal lobes move in to wrap around the hydrophobic residues of a target molecule [10], [11]. Besides this classical mode of binding, CaM bound to oedema factor adopts an extended conformation [12]. In other cases, part of the central α-helix transforms into loops to facilitate peptide interactions [13], [14], or binding to the target peptide occurs only *via* the C-terminal lobe [15], [16]. Moreover, NMR and other spectroscopic studies have shown that the central α-helix is flexible in solution, and the backbone atoms between residues Lys77-Asp80 undergo conformational changes [17]. Although CaM is best characterized to specifically bind with Ca^2+^, a number of studies have indicated that it can also bind with other metal ions [18], [19], [20]. Besides, CaM is able to selectively bind Ca^2+^ despite the fact that, in the resting cell, there are high levels of other cations, especially magnesium (Mg^2+^), which is present in roughly a 10^2^–10^4^-fold higher concentration than intracellular Ca^2+^ [21]. Zinc (Zn^2+^) antagonizes the calcium action by inhibiting the same cellular reactions triggered by Ca^2+^. This inhibition occurs through binding of Zn^2+^ to the CaM-protein complex [22].

The calpacitin protein family members, neuromodulin (Nm) and neurogranin (Ng), are intrinsically unstructured proteins, and are shown to interact with *apo* CaM (Ca^2+^ free). In the present study, the IQ peptides derived from Nm and Ng were present in the crystallization drops; however, no electron density was observed to confirm the existence of complex in the crystal. This crystal structure of CaM, in which no substrate peptide was bound (hereafter referred as ligand-free CaM), adopts a unique *trans* conformation that has so far not been observed.

## Materials and Methods

### CaM Expression and Purification

The *CaM* (accession no NP_033920) gene was cloned into the pETDuet-1 expression vector (Novagen, Madison, WI) at the cloning site 1, following standard procedures. The recombinant plasmid (pETDuet-CaM) was transformed into chemically competent *E. coli BL21 (DE3)* cells for the large-scale protein production. The cells were grown in 1 L of LB media, and the cell pellet was obtained by centrifugation at 9800 g. Cell pellets were re-suspended in 100 ml of lysis buffer composed of 50 m*M* Tris-HCl (pH-8.0), 500 m*M* NaCl, 10% v/v glycerol, 20 m*M* imidazole, 20 m*M* BME and 0.1 m*M* PMSF, and lysed by sonication. The cell lysate was centrifuged at 39,000 g for 30 min at 4°C. The supernatant was mixed with 5 ml of Ni–NTA resin (Qiagen, Valencia, CA) for 2 h at 4°C. Ni–NTA resin was washed thrice using 10 ml of lysis buffer each time. Subsequently, the bound proteins were eluted from Ni-NTA beads using lysis buffer supplemented with 300 m*M* imidazole (pH 8.0). Next, the eluted CaM was passed through the gel filtration column, HiLoad™ 16/60 Super- dex™ 75 prep grade (Amersham Biosciences, Uppsala, Sweden) in a buffer containing 20 m*M* HEPES (pH 8.0), 100 m*M* NaCl and 10 m*M* CaCl_2_. The protein was concentrated up to 12 mg/ml and taken for crystallization experiments. The selenomethionine-labeled proteins were produced using LeMaster media [23] following a similar procedure as described above. All protein purification steps were carried out at 4°C, unless otherwise stated.

### Crystallization and Structure Determination

The commercially synthesized IQ motifs of Nm (^34^AATKIQASFRGHITRKKLKGEKKG^57^) and Ng (^27^NAAAAKIQASFRGHMARKKIKSGE^50^) were present in the crystallization mixture in the molar ratio of 1∶3 (CaM: peptide). The initial objective of this work was to crystallize CaM as a complex with these peptides. However, we obtained only the crystals of CaM. The crystallization experiments were conducted at room temperature using hanging drop vapor diffusion method. The initial crystallization conditions obtained from PACT Suite (Qiagen) were further optimized. The diffraction quality crystals of ligand-free CaM were obtained using a reservoir solution consisting of 100 m*M* Tris-HCl (pH 8.0), 8–10% v/v PEG 6 K, 5–10 m*M* ZnCl_2_ and 10% v/v glycerol. Crystals were directly picked from drop and flash-cooled in N_2_ cold stream at 100 K.

Brief attempts with molecular replacement method did not yield any structure solution; this led us to collect the Single-wavelength Anomalous Dispersion (SAD) dataset in the synchrotron beam line X8C (NSLS, Brookhaven National Laboratory, Upton, New York) using a Quantum 4-CCD detector (Area Detector Systems Corp Poway, CA, USA) and processed using HKL2000 [24]. Heavy atom (Se) location, phasing and density modification were performed using the program ShelxC/D/E [25], and model building was carried out with the program Buccaneer [26] in CCP4. When necessary, the model was manually built in COOT [27] and refinement was performed in Refmac5 [28]. At the final stage of refinement, well-ordered water molecules were included. The model had good stereochemistry, as analyzed by PROCHECK [29] (Table 1). All structure-related figures reported in this manuscript were prepared using PyMol [30].

**Table 1:** Data collection and refinement statistics.

	Ca^2+^/CaMPeak
Cell parameters (Å, °)	a = 38.83,b = 116.40,c = 38.80,β = 94.83
Space group	P2_1_
**Data collection**	
Resolution range (Å) *	50.00–2.02 (2.05–2.02)
Wavelength (Å)	0.978
Observed reflections >1σ	165195
Unique reflections	45014 (2341)
Completeness (%)	99.3 (98.9)
Overall (I/σ (I))	34.6 (5.2)
R_sym_ a (%)	5.5 (38.1)
Matthew Coefficient (V_M,_ A^3^/Da)(Solvent %)	2.5(50.73)
**Refinement and quality** b	
Resolution range (Å)	28.58–2.00
R_work_ c (no. of reflections)	0.20 (21561)
R_free_ d (no. of reflections)	0.23 (1191)
RMSD bond lengths (Å)	0.014
RMSD bond angles(^o^)	1.760
No atoms	
Protein	2208
Ions	10
Water	80
Average B-factors^e^ (Å^2^)	
Main chain	34.25
Side chainWaters	39.8646.70
**Ramachandran plot**	
Most favoured regions (%)	92.2
Additional allowed regions (%)	7.0
Generously allowed regions (%)	0.8
Disallowed regions (%)	0.0

a R_sym_ = ∑|I_i_ – <I>|/|I_i_| where I_i_ is the intensity of the i^th^ measurement, and <I> is the mean intensity for that reflection.

b Reflections with I>σ was used in the refinement.

c R_work_ = |F_obs_ – F_calc_|/|F_obs_| where F_calc_ and F_obs_ are the calculated and observed structure factor amplitudes, respectively.

d R_free_ = as for R_work_, but for 5–7% of the total reflections chosen at random and omitted from refinement.

e Individual B-factor refinements were calculated.

* The high resolution bin details are in the parenthesis.

### Protein Data Bank Accession Code

Coordinates and structure factors of the ligand-free Ca^2+^/Zn^2+^/CaM has been deposited with RCSB Protein Data Bank with codes 4HEX.

## Results and Discussion

### Overall Structure

**Figure 1:**
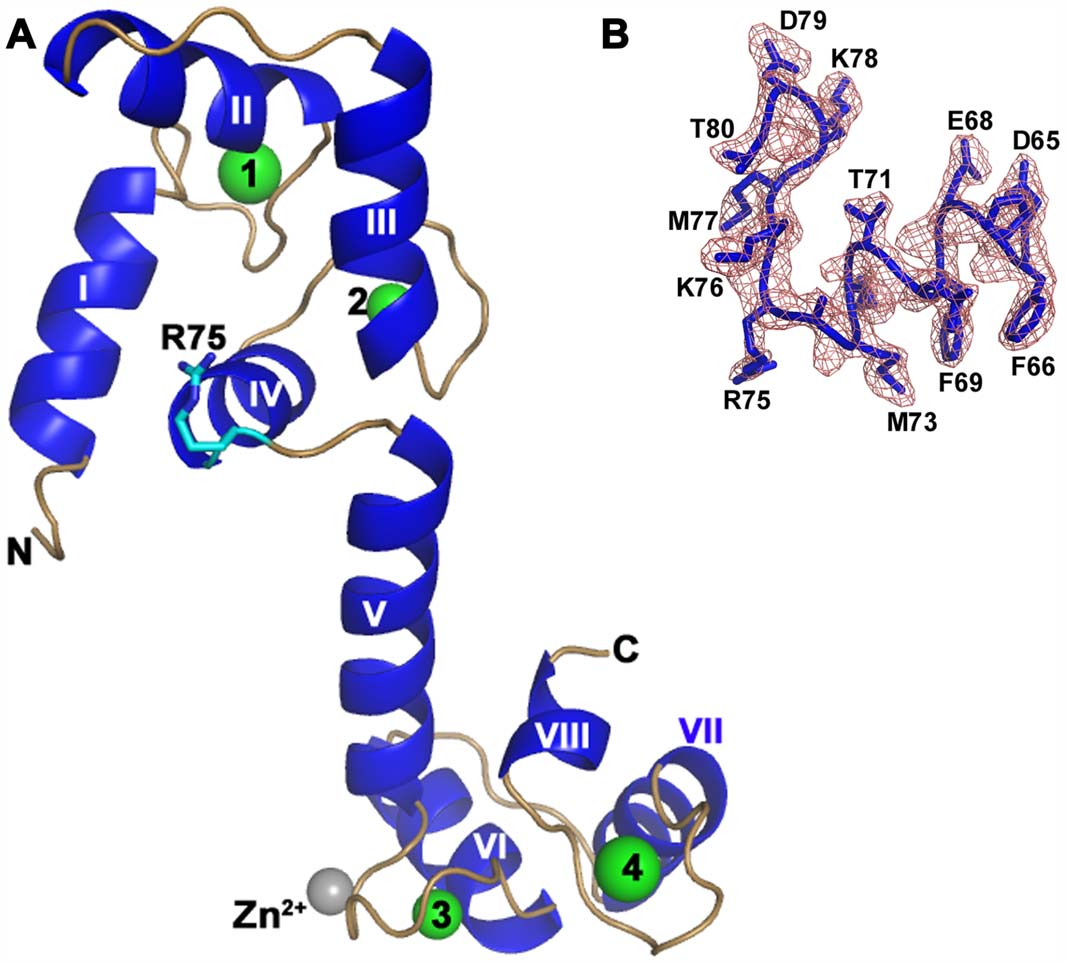
A novel *trans* conformation of calmodulin (CaM). **A:** Ribbon representation of novel conformation of ligand-free CaM. The helices, loops, Ca^2+^ and Zn^2+^ are shown in blue, pale green, green and grey, respectively. The CaM molecule adopts an extended dumbbell-shaped conformation and the two domains are well separated. The bending at Arg75 is shown in stick representation. The α-helices are numbered from I-VIII. **B:** *2Fo-Fc* electron density map for the region 65–80 aa of CaM. This map is contoured at a level of 1σ.

The crystal structure of ligand-free CaM was determined by Single-wavelength Anomalous Dispersion (SAD) method and refined to an R-factor of 0.20 (R_free_ = 0.23) up to 2.02 Å resolution. The electron density for the N-terminal His-tag and the first 5 residues were not well-defined and were therefore not included in the model. The model had good geometry, with no residues in the disallowed region of the Ramachandran plot (Table 1). There are two identical molecules (RMSD 0.7 Å) in the asymmetric unit (Supplementary Figure S1). However, the *PISA* server [31] analysis and gel filtration chromatography suggest that ligand-free CaM is a monomer. The Ca^2+^ ions were well-defined in the electron density map, located at each EF-hand motif. Besides a Zn^2+^ ion was observed near His108 for the first time in CaM. The ligand-free CaM molecule was in an open conformation, wherein helices of EF motifs were perpendicular to each other (Figure 1).

### Calmodulin Adopts a Novel Conformation

**Figure 2:**
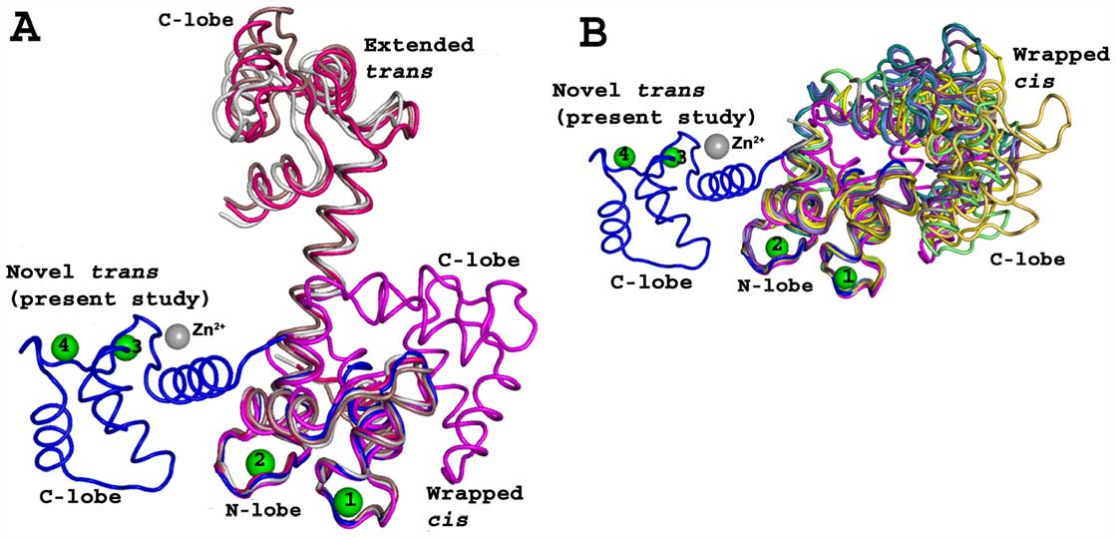
Comparison among various calmodulin (CaM) structures. **A:** The Cα superposition of the present study novel *trans* conformation of CaM with several extended (*trans*) CaM conformations: 1PRW (magenta), 2F2P (white), 2W73 (red), and 3CLN (dark salmon). **B:** The Cα superposition of present study novel *trans* conformation of CaM with several wrapped (cis) CaM conformations: 2BE6 (yellow), 2F3Y (light blue), 2O60 (pale green), 2VAY (teal), 2X0G (orange), 3BXK (deep purple), 3DVE (gray), 1CDM (olive). CaM conformations can be classified as “wrapped” and “extended”. In “wrapped” conformation, the two lobes are close to each others in *cis* orientation. In “extended” conformation, the two lobes are widely separated in *trans* orientations. In the present study, CaM adopted a novel *trans* conformation. The positions of metal ions in the current structure (blue) are labeled as Ca^2+^ (Green) and Zn^2+^ (grey). These structure alignments were carried out in PyMol [30].

**Figure 3:**
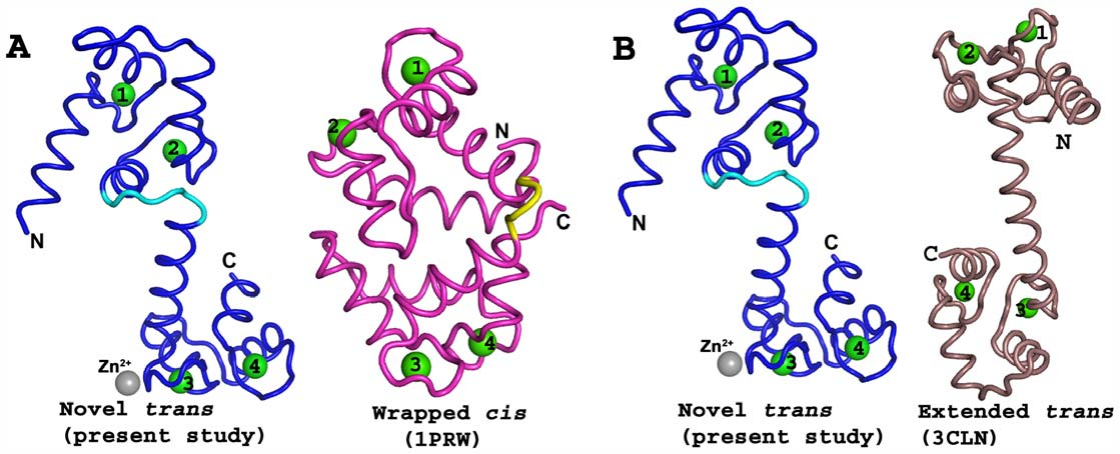
Comparison of *cis* and *trans* conformations of Calmodulin. **A:** Side-by-side comparison of the novel *trans* (current structure, blue) and *cis* (pdb code 1PRW, magenta) conformations of CaM, which show the unwinding region of central helix in both structures in cyan and yellow, respectively. **B:** Side-by-side comparison of the novel *trans* (current structure, blue) and extended *trans* (pdb code 3CLN, dark salmon) conformations of CaM. In the extended *trans* conformation of CaM, no unwinding of the central helix was observed. The positions of the metal ions in the current novel *trans* (blue) are labeled as Ca^2+^ (Green) and Zn^2+^ (grey). In wrapped *cis* (1PRW, magenta) and extended *trans* (3CLN, dark salmon), all sites (EF1-EF4) were occupied by Ca^2+^ (Green).

A search for structural homologs in the PDB database using the DALI program [32] did not identify any similar structures. In the present structure, the ligand-free CaM adopts a unique conformation, with the relative disposition of the two lobes being completely different to any of the previously reported CaM structures (Figure 2). However, a one-to-one comparison of individual domains showed no significant structural differences (rmsd less than 0.8 Å for all Cα atoms). The structurally similar two lobes were in *trans* conformation about the axis of the central helix (Figures 2 and 3).

A close examination of the current ligand-free CaM structure with previously reported CaM complexes showed that residues Ala74-Asp79 of the central helix (aa 65–92) are unwound, and bent by ∼90° near Arg75; this reoriented the C-lobe in a perpendicular direction to the central helix (Figure 1 and 2). While a transformation of α-helix to loops has been previously reported [13], [14], the kink observed at Arg75 is unique. This unique structure of CaM represents one of its many possible conformations. The side chain of Arg75 is exposed on the surface of the molecule, which makes four hydrogen bonding contacts only with symmetry-related molecules (Arg38 and Arg127). The B-factors are the indicators of ordered nature of the atoms. The average B-factors for Arg75 of chain A and B are 47.5 and 50.9 Å^2^, respectively. These average values are in good agreement with the B factors of the neighbouring residues, and suggest that Arg75 is well ordered (Figure 1). A detailed study of Lys76 mutation on CaM conformation was carried out by Medvedeva *et al.* [33]. A double mutation, containing a KGK insertion between residues 81 and 82, and a point mutation of K76P, makes the central helix highly flexible in Ca^2+^/CaM, as determined by the trypsinolysis [33]. Two mutants (K76P and K76E) were regarded as having a distorted central helix, and showed high resistance to trypsinolysis in the absence of Ca^2+^ [33]. Mutants K76A and K76V, on the other hand, decreased the rate of trypsinolysis of the central helix with a simultaneous increase in the rate of trypsinolysis in the C-terminal domain of CaM [33]. These studies revealed that various mutations in the central helix alter the conformation of CaM and confirm the highly flexible nature of the central helix, as observed through NMR studies [17].

Previously, a closed, compact crystal structure of CaM (PDB 1PRW) was reported at 1.7 Å resolution [34]. In this structure, CaM existed in a compact ellipsoidal conformation and revealed a sharp bend in the central helix. The two lobes were in *cis* orientation, in contrast to the *trans* orientation observed in the current study. The N-lobe and C-lobe were close to each other and made several inter-domain contacts (Figure 3). The residues Asp79-Ser82 were unwound and made a type 1 reverse turn [34]. The complex structure of CaM and IQ peptide from Lc-, P/Q-, and R-type voltage-dependent Ca^2+^ channels is similar, wherein the central helix of CaM unwinds and peptides are wrapped by two lobes of CaM ( [35], [36]; 3BXK and 3BXL). Similarly, the structure of *apo* CaM bound to the first two IQ motifs of the murine myosin V heavy chain adopts a unique conformation, in which central helix unwinds and the N and C lobes wrap around the peptides [37].

All the above CaM complex structures adopt a *cis* conformation. However, in the current study, the conformation of CaM was in an extended form, and the two globular lobes were widely separated. As noted above, residues Ala74-Asp79 were unwound by one turn and formed a sharp bend at Arg75. However, incubating Nm/Ng or their IQ peptides with Ca^2+^/CaM did not show any spectral changes to indicate any conformational change in CaM [38]. Nonetheless, the observed conformation of CaM in this study represents a novel *trans* structure of CaM. The implication of this conformation is yet to be studied. The bending of the central helix is a key feature of the conformational dynamics of CaM in recognizing the target [9].

**Figure 4:**
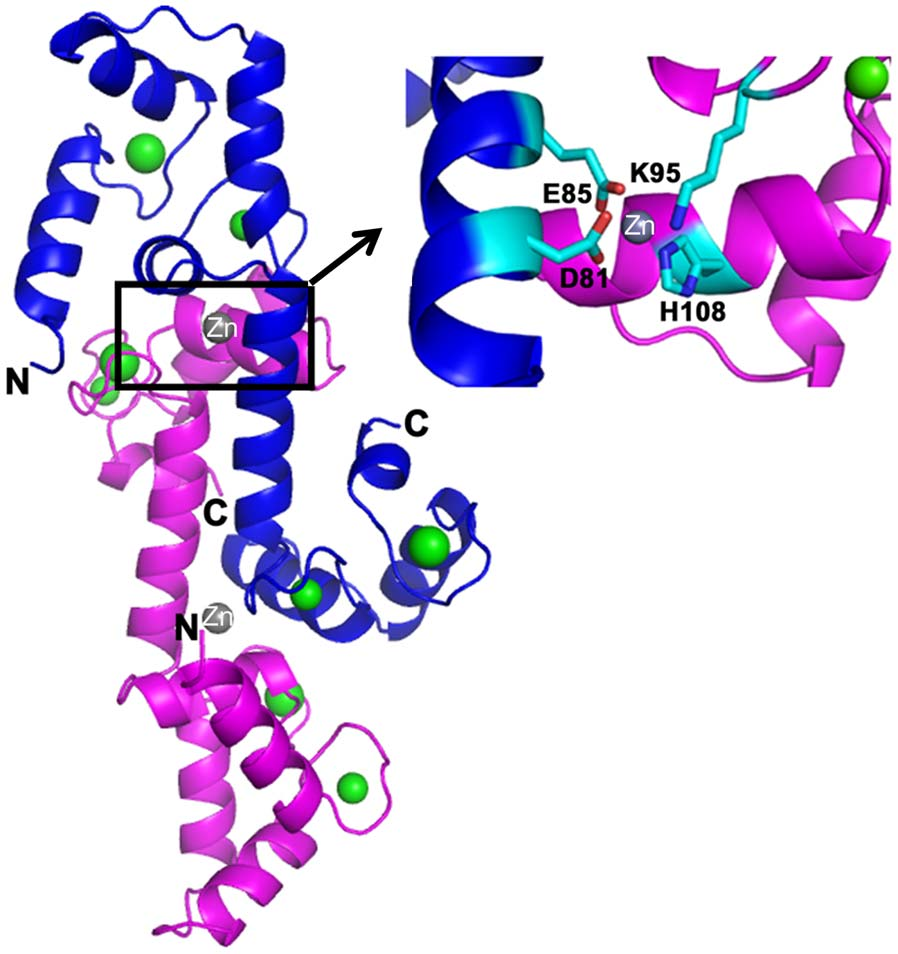
Coordination of Zn^2+^ ion: A Zn^2+^ ion (grey) bound to His108 and Lys95 of chain A (magenta), and Asp81 and Glu85 from chain B (blue). A similar Zn^2+^ ion is also present in chain A.

It has to be noted that the Ca^2+^/CaM (buffer supplemented with 10 m*M* CaCl_2_) crystals were grown in conditions containing 5–10 m*M* ZnCl_2_. A previous flow dialysis study showed that CaM has two higher (80–300 µM) affinity Zn^2+^ ion sites and four or five lower affinity Zn^2+^-binding sites [22], [39]. Nevertheless, the previously reported Zn^2+^-bound, N-lobe CaM structure resembles the *apo* CaM structures (i.e. the closed form of CaM) [39]; by comparison, CaM adopts an open form in the current structure. Further, no similar bend at Arg75 was observed in the previous structure despite Zn^2+^ binding to both EF-hand motifs in the N-lobe [39]. Based on the heavy atom peak heights in the anomalous map and the refinement statistics comparison (R-values and B-factors) we assigned the observed electron density as Ca^2+^ ions in the EF-hand motifs and a Zn^2+^ ion near His108 (Figure 4). However the possibility of having less occupancy Zn^2+^ that might mimic a fully occupied Ca^2+^ cannot be ruled out. We have observed that lowering the ZnCl_2_ concentration in crystallization conditions reduces the nucleation and results in good quality crystals. The observed Zn^2+^ ion at chain A is coordinating with His108, Lys95 (chain A), Asp81 and Glu81 (chain B) and *vice versa* for chain B. A similar coordination for Zn^2+^ ion has been reported in ALE-1, a glycylglycine endopeptidase from *Staphylococcus capitis EPK1* [40].

In CaM, Ca^2+^ binding occurs sequentially, first in the C-lobe followed by N-lobe binding. C-lobe has much higher affinity for Ca^2+^ than does the N-lobe. Ca^2+^ binding to CaM rearrange the EF motifs in each lobe, central helix becomes α helical but no such bend has been observed [6], [41], [42]. Previously, the Ca^2+^ in Ca^2+^/CaM crystals was replaced by Pb^2+^ and Ba^2+^ by soaking. The crystal structures of Pb^2+^/CaM and Ba^2+^/CaM did not show large conformational changes as compared with Ca^2+^/CaM [43], [44]. Thus, the present conformational change observed in the central helix of the CaM is independent of the bound metal ions. The large conformational changes in proteins are often associated with ligand/partner binding. One proposed function for Nm and Ng is to sequester CaM at the membrane in the vicinity of ‘CaM-activated enzymes’ under low Ca^2+^ conditions at the pre- and post-synaptic terminals, respectively. Elevations of intracellular free Ca^2+^ would promote dissociation of CaM from Nm and Ng [45]. We speculate that upon Ca^2+^ binding to CaM-Nm/Ng, CaM might undergo some conformational change, similar to the one reported here, to release Nm/Ng. This has to be approached cautiously and warrants experimental verification.

In summary, CaM is known to interact with over 100 different proteins to modulate their activity, adopting various conformations to engage with its binding partners. In the present study no electron density for the IQ peptide was observed to confirm the existence of its complex in the crystal; thus, only the ligand-free CaM was crystallized. The observed ∼90° bend at the central α-helix near Arg75 may represent a key conformational dynamics of CaM essential for engaging its target. This study reveals a novel *trans* conformation of CaM as one of many possible conformations that has so far not been observed.

## Supporting Information

Figure S1 **This diagram shows the packing of the symmetry-related molecules in the crystal.** The two molecules of the asymmetric unit were shown in blue and magenta respectively. The nearest symmetry related molecules shown in different colors.
(TIF)
