# Supplementary figures and images for: A Novel Trans Conformation of Ligand-Free Calmodulin
Source: PLoS One. 2013 Jan 29;8(1):e54834. doi: 10.1371/journal.pone.0054834 (PMC3558517; doi:10.1371/journal.pone.0054834)

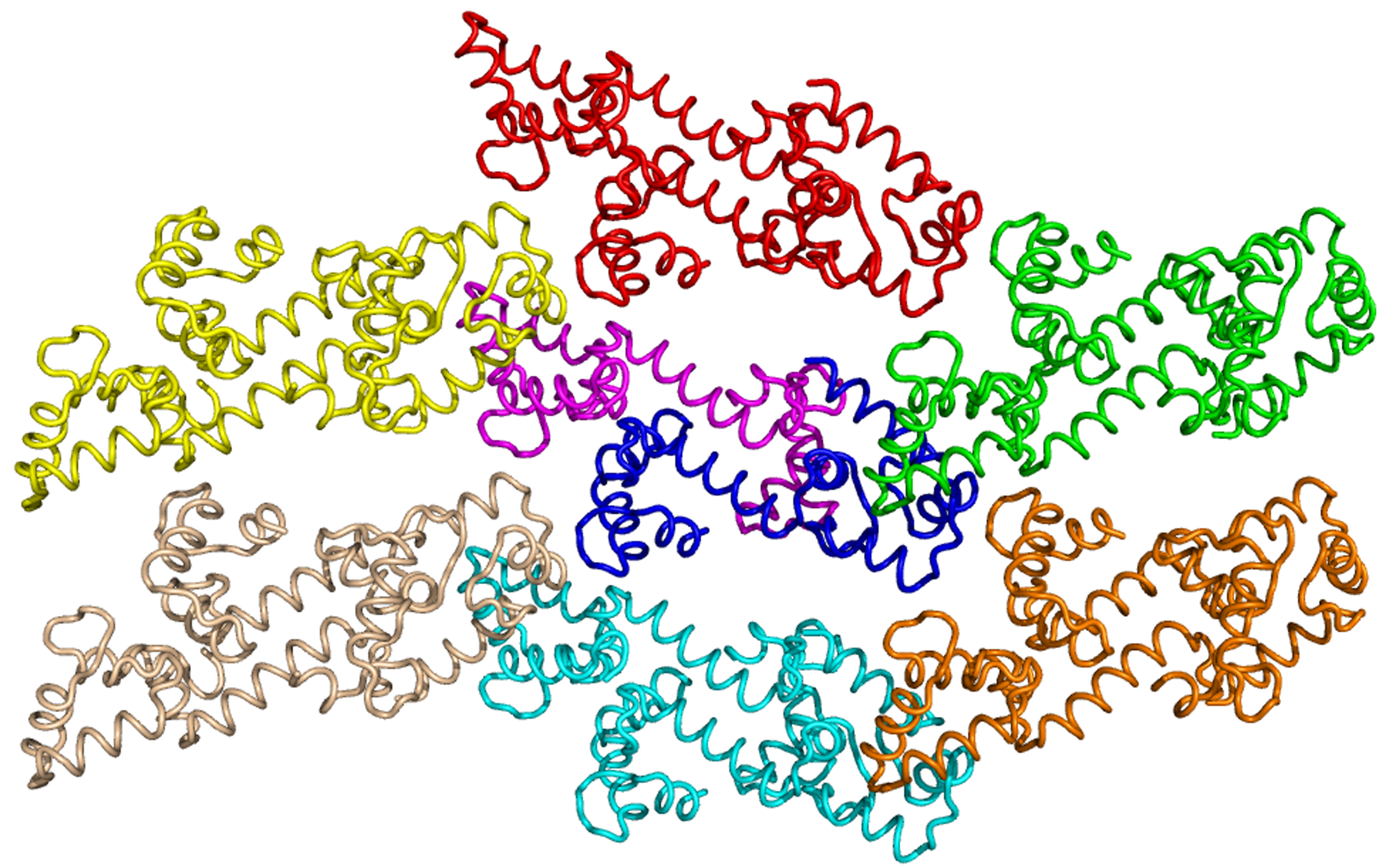

Supplement: Figure S1 — This diagram shows the packing of the symmetry-related molecules in the crystal. The two molecules of the asymmetric unit were shown in blue and magenta respectively. The nearest symmetry related molecules shown in different colors. (TIF) [file pone.0054834.s001.tif]
